# Negative heterosis for meiotic recombination rate in spermatocytes of the domestic chicken Gallus gallus

**DOI:** 10.18699/VJ21.075

**Published:** 2021-10

**Authors:** L.P. Malinovskaya, K.V. Tishakova, T.I. Bikchurina, A.Yu. Slobodchikova, N.Yu. Torgunakov, A.A. Torgasheva, Y.A. Tsepilov, N.A. Volkova, P.M. Borodin

**Affiliations:** Institute of Cytology and Genetics of the Siberian Branch of the Russian Academy of Sciences, Novosibirsk, Russia; Novosibirsk State University, Novosibirsk, Russia; Novosibirsk State University, Novosibirsk, Russia; Institute of Molecular and Cellular Biology of the Siberian Branch of the Russian Academy of Sciences, Novosibirsk, Russia; Institute of Cytology and Genetics of the Siberian Branch of the Russian Academy of Sciences, Novosibirsk, Russia; Novosibirsk State University, Novosibirsk, Russia; Institute of Cytology and Genetics of the Siberian Branch of the Russian Academy of Sciences, Novosibirsk, Russia; Novosibirsk State University, Novosibirsk, Russia; Institute of Cytology and Genetics of the Siberian Branch of the Russian Academy of Sciences, Novosibirsk, Russia; Novosibirsk State University, Novosibirsk, Russia; Institute of Cytology and Genetics of the Siberian Branch of the Russian Academy of Sciences, Novosibirsk, Russia; Novosibirsk State University, Novosibirsk, Russia; Institute of Cytology and Genetics of the Siberian Branch of the Russian Academy of Sciences, Novosibirsk, Russia; Novosibirsk State University, Novosibirsk, Russia; L.K. Ernst Federal Research Center for Animal Husbandry, Dubrovitsy, Moscow region, Russia; Institute of Cytology and Genetics of the Siberian Branch of the Russian Academy of Sciences, Novosibirsk, Russia; Novosibirsk State University, Novosibirsk, Russia

**Keywords:** recombination;, heterosis;, macrochromosomes, synaptonemal complexes, MLH1., рекомбинация, гетерозис, макрохромосомы, синаптонемные комплексы, MLH1

## Abstract

Benef its and costs of meiotic recombination are a matter of discussion. Because recombination breaks allele combinations already tested by natural selection and generates new ones of unpredictable f itness, a high recombination rate is generally benef icial for the populations living in a f luctuating or a rapidly changing environment and costly in a stable environment. Besides genetic benef its and costs, there are cytological effects of recombination, both positive and negative. Recombination is necessary for chromosome synapsis and segregation. However, it involves a massive generation of double-strand DNA breaks, erroneous repair of which may lead to germ cell death or various mutations and chromosome rearrangements. Thus, the benef its of recombination (generation of new allele combinations) would prevail over its costs (occurrence of deleterious mutations) as long as the population remains suff iciently heterogeneous. Using immunolocalization of MLH1, a mismatch repair protein, at the synaptonemal complexes, we examined the number and distribution of recombination nodules in spermatocytes of two chicken breeds with high (Pervomai) and low (Russian Crested) recombination rates and their F1 hybrids and backcrosses. We detected negative heterosis for recombination rate in the F1 hybrids. Backcrosses to the Pervomai breed were rather homogenous and showed an intermediate recombination rate. The differences in overall recombination rate between the breeds, hybrids and backcrosses were mainly determined by the differences in the crossing over number in the seven largest macrochromosomes. The decrease in recombination rate in F1 is probably determined by diff iculties in homology matching between the DNA sequences of genetically divergent breeds. The suppression of recombination in the hybrids may impede gene f low between parapatric populations and therefore accelerate their genetic divergence.

## Abstract (ru)

Преимущества и издержки мейотической рекомбинации являются предметом дискуссий. По- скольку рекомбинация разрушает комбинации аллелей, уже проверенные естественным отбором, и порож- дает новые с непредсказуемой приспособленностью, высокая частота рекомбинации обычно выгодна для популяций, живущих в быстро меняющейся среде, но не выгодна в стабильной среде. Помимо генетических преимуществ и издержек, существуют цитологические эффекты рекомбинации, как положительные, так и отрицательные. Рекомбинация необходима для синапсиса и сегрегации хромосом. Однако она сопряжена с образованием множества двухцепочечных разрывов ДНК, ошибочная репарация которых может привести к гибели половых клеток или к различным мутациям и перестройкам хромосом. Таким образом, преимущества рекомбинации (генерация новых комбинаций аллелей) будут преобладать над ее издержками (возник- новение вредных мутаций), пока популяция остается достаточно гетерогенной. Используя иммунолокали- зацию MLH1 белка мисматч-репарации, мы исследовали количество и распределение рекомбинационных узелков в сперматоцитах двух пород кур с высокой (Первомайская) и низкой (Русская хохлатая) частотой рекомбинации и их гибридов F1 и беккроссов. У гибридов F1 мы наблюдали отрицательный гетерозис по частоте рекомбинации. Беккроссы на Первомайскую породу были достаточно однородными и имели про- межуточную частоту рекомбинации. Различия в общей частоте рекомбинации между породами, гибридами и беккроссами в основном определялись различиями по макрохромосомам. Снижение частоты рекомби- нации в F1, вероятно, обусловлено трудностями в поиске гомологии между последовательностями ДНК ге- нетически дивергентных пород. Подавление рекомбинации у гибридов может препятствовать потоку генов между парапатрическими популяциями и, следовательно, ускорять их генетическую дивергенцию.

## Introduction

Benefits and costs of meiotic recombination are a favorite subject of theoretical discussions and mathematical models (Kondrashov, 1993; Otto, Lenormand, 2002; Hartfield, Keightley, 2012; Rybnikov et al., 2020). They are mostly focused on the population genetic effects of recombination, i. e. its contribution to genetic and phenotypic variability. Crossing over reduces linkage disequilibrium by breaking old allele combinations already tested by natural selection and generating new ones of unpredictable fitness. Therefore, a high recombination rate is generally beneficial for populations living in fluctuating or rapidly changing environments and costly in a stable environment (Otto, Michalakis, 1998; Lenormand, Otto, 2000). Besides genetic benefits and costs, there are cytological effects of recombination, both positive and negative. Recombination is necessary for chromosome synapsis and segregation. However, it involves a massive generation of double-strand DNA breaks. Insufficient or erroneous repair of the breaks leads to the death of the affected germ cells or various mutations and chromosome rearrangements (Zickler, Kleckner, 2015).

Crossing over distribution along the chromosomes is another important variable affecting both genetic and cytological benefits and costs of recombination. Two crossing overs positioned too close to each other do not affect the linkage phase (Gorlov, Gorlova, 2001; Berchowitz, Copenhaver, 2010). Similarly, crossing overs located too close to a centromere of an acrocentric chromosome or to telomere do not produce new allele combinations. In these cases, the cost of recombination is paid, but no benefit is gained. Cytological costs of crossing overs that are too distal or too proximal should also be taken into account. They often lead to incorrect chromosome segregation and generation of chromosomally unbalanced gametes (Koehler et al., 1996; Hassold, Hunt, 2001). Thus, the benefits of recombination (generation of new allele combinations) would prevail over its costs (occurrence of deleterious mutations) as long as the population remains sufficiently heterogeneous.

The heritability of recombination rate was estimated as 0.30 in humans, 0.22 to 0.26 in cattle and 0.15 in sheep (Kong et al., 2004; Sandor et al., 2012; Johnston et al., 2016). Interbreed variation in recombination rate was detected in rams (Davenport et al., 2018) and roosters (Malinovskaya et al., 2019). The most intriguing finding of the latter study was a correspondence between the age of the breed and its recombination rate. Relatively young breeds created by crossing several local breeds showed high recombination rates, while ancient local breeds displayed a low recombination rate. The decrease in recombination rate with breed age might be a correlative response to a decrease in genetic heterogeneity within each breed with time due to inbreeding and artificial selection (Lipinski et al., 2008; Gibbs et al., 2009). Early stages of conscious selection for economic traits were probably accompanied by unconscious selection for a high recombination rate. A reduction of genetic variability, an inevitable result of inbreeding and selection, leads to a decrease in recombination efficiency and therefore reduces selective advantages of high recombination rate

In this paper, we examine the inheritance of the recombination rate in male F1 hybrids and backcrosses of the chicken breeds showing the highest (Pervomai) and lowest (Russian Crested) level of recombination among the six breeds examined by L.P. Malinovskaya et al. (2019). The Pervomai breed was produced in 1930–1960 by a complex reproductive crossing of three crossbred breeds: White Wyandotte (derived from crosses between Brahmas and Hamburgs), Rhode Island (derived from crosses between Malays and brown Italian Leghorns) and Yurlov Crower (derived from crosses of Chinese meat chicken, gamecocks and landraces). Russian Crested is an ancient local breed described in the European part of Russia in the early XIX century (Paronyan, Yurchenko, 1989).

We estimated the number and distribution of recombination nodules in spermatocytes using immunolocalization of MLH1, a mismatch repair protein of mature recombination nodules, at the synaptonemal complexes (SCs). This method has proved to produce reliable estimates of the overall recombination frequency and the distribution of recombination events along individual chromosomes (Anderson et al., 1999; Froenicke et al., 2002; Segura et al., 2013; Pigozzi, 2016).

## Material and methods

Animals. Thirty-four adult five-month-old roosters were used in this study. Eight of them were Pervomai breed, nine – Russian Crested breed, three – F1 hybrids between Pervomai dams and Russian Crested sires, fourteen – backcrosses of F1 sires to Pervomai dams

The roosters were bred, raised and maintained at the poultry farm of the L.K. Ernst Federal Research Centre for Animal Husbandry under conventional conditions. Maintenance, handling and euthanasia of animals were carried out in accordance with the approved national guidelines for the care and use of laboratory animals. All experiments were approved by the Ethics Committee on Animal Care and Use at the Institute of Cytology and Genetics of the Siberian Branch of the Russian Academy of Sciences (approval No. 35 of October 26, 2016 and 45/2 of January 10, 2019).

Synaptonemal complex spreading and immunostaining. Chromosome spreads were prepared from the right testes by a drying-down method (Peters et al., 1997). Then the slides were subjected to immunostaining according to L.K. Anderson et al. (1999). The slides were incubated overnight in a humid chamber at 37 °C with the following primary antibodies: rabbit polyclonal anti-SYCP3 (1:500; Abcam, Cambridge, UK), mouse monoclonal anti-MLH1 (1:30; Abcam, Cambridge, UK) and human anticentromere (ACA) (1:70; Antibodies Inc., Davis, USA). Secondary antibody incubations were carried out for 1 h at 37 °C. The secondary antibodies used were Cy3- conjugated goat anti-rabbit (1:500; Jackson ImmunoResearch, West Grove, USA), fluorescein isothiocyanate (FITC)-conjugated goat anti-mouse (1:30; Jackson ImmunoResearch, West Grove, USA) and aminomethylcoumarin (AMCA)-conjugated donkey anti-human (1:40; Jackson ImmunoResearch, West Grove, USA).

Antibodies were diluted in PBT (3 % bovine serum albumin and 0.05 % Tween 20 in PBS). A solution of 10 % PBT was used for blocking non-specific binding of antibodies. Vectashield antifade mounting medium (Vector Laboratories, Burlingame, CA, USA) was used to reduce fluorescence fading. The preparations were visualized with an Axioplan 2 microscope (Carl Zeiss, Germany) equipped with a CCD camera (CV M300, JAI Corporation, Yokohama, Japan), CHROMA filter sets and ISIS4 image-processing package (MetaSystems GmbH, Altlußheim, Germany). The location of each imaged immunolabeled SC spread was recorded so that it could be relocated on the slide after FISH

Fluorescence in situ hybridization with BAC probes. After the acquisition of the immunofluorescence signals, the slides were subjected to FISH with universal bird BAC probes CHORY-261 (Damas et al., 2017). Table shows a list of BAC-clones used in this study. BAC DNA was isolated using the Plasmid DNA Isolation Kit (BioSilica, Novosibirsk, Russia) and amplified with GenomePlex Whole Genome Amplification Kit (Sigma-Aldrich Co., St. Louis, MO, USA). BAC DNA was labeled using GenomePlex WGA Reamplification Kit (Sigma-Aldrich Co.) by incorporating biotin-16-dUTP (Roche, Basel, Switzerland).

**Table.**
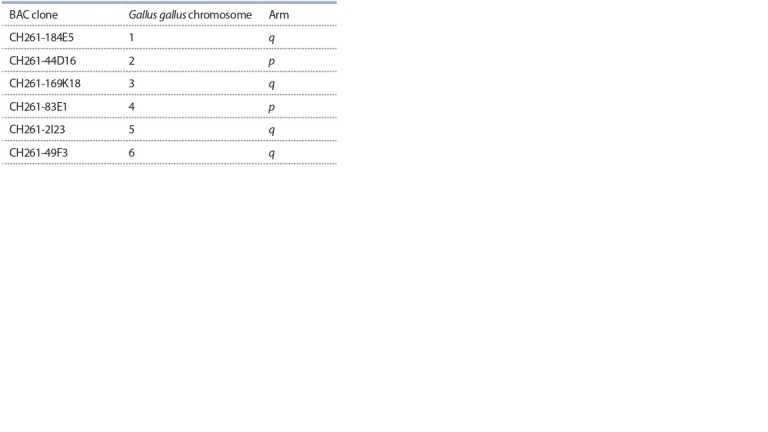
List of BAC clones used for FISH

FISH on SCs was performed following the standard procedure (Liehr et al., 2017). Briefly, 16 μl of hybridization mix contained 0.2 μg of the labeled BAC-probe, 2 μg of Cot-2 DNA of Gallus gallus (Trifonov et al., 2009), 50 % formamide in 2xSSC (saline-sodium citrate buffer), 10 % dextran sulfate. Probes were denatured for 5 min at 95 °C and reannealed for 1 h at 42 °C. Synaptonemal complexes spreads were denatured in 70 % formamide in 2xSSC for 3 min at 72 °C. Hybridization was made overnight at 42 °C. Posthybridization washes included 2×SSC, 0.4×SSC, 0.2×SSC (5 min each, 60 °C) followed by 20-min incubation in 4 % dry milk in 4×SSC/0.05 % Triton X-100. All washes were performed at 42 °C in 4×SSC/0.05 % Triton X-100 3 times (5 min each). Hybridization signals were detected with fluorescein avidin DCS and biotinylated anti-avidin D (Vector Laboratories, Inc.).

Image analysis. We measured the length of each SC and the total SC length in μm, scored the number of MLH1 signals localized on SCs and recorded their positions relative to the centromere using MicroMeasure 3.3 software (Reeves, 2001). For the seven largest macroSCs identified by relative lengths and centromeric indices, we visualized the pattern of MLH1 foci distribution. We divided the average length of SC by intervals and plotted the relative number (the proportion) of MLH1 foci within each interval. To make the intervals on chromosomes of different lengths comparable, we set the number of intervals for each SC proportional to the average SC length, being ~1 μm.

The Statistica 6.0 software package (StatSoft) was used for descriptive statistics. Mann–Whitney U-test was used to estimate the differences between the genotypes in the average number of MLH1 foci per cell and each macrochromosome, p < 0.01 was considered to be statistically significant. Values in the text and figures are presented as means ± S.D.

## Results

**Fig. 1.**
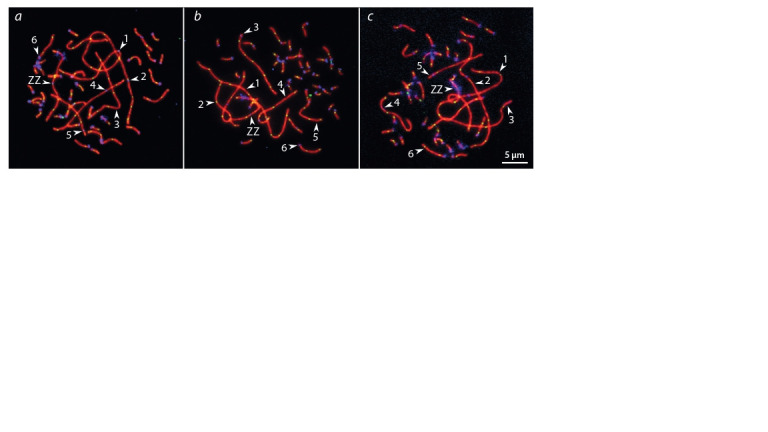
Pachytene spermatocytes of Pervomai (a) and Russian Crested (b) and backcross (c) roosters after immunolocalization of SYCP3 (red), centromeric proteins (blue) and MLH1 (green). Arrowheads point to the SCs of the macrochromosomes identif ied by their lengths and centromeric indices.

We analyzed the number and distribution of MLH1 foci at 52 650 SC in 1350 spermatocytes of 34 roosters. The rooster pachytene karyotype contained 38 autosomal SCs and a ZZ pair. We identified the seven largest macroSCs by their relative lengths and centromeric indices. SC1, SC2 and SCZZ were large metacentrics. They differed from each other in length and centromeric indices ( p < 0.001). SC3 and SC5 were large and medium-sized acrocentics, while SC4 and SC7 were medium-sized submetacentics, which also differed from each other in their relative lengths and centromeric indices. The macroSCs 6, 8–10 and all microSCs were acrocentric, with gradually decreasing chromosomal sizes (Fig. 1). All chromosomes showed orderly synapsis. No SCs with asynapsis were detected at pachytene spreads of the specimens of the parental breeds and their F1 hybrids and backcrosses

**Fig. 2.**
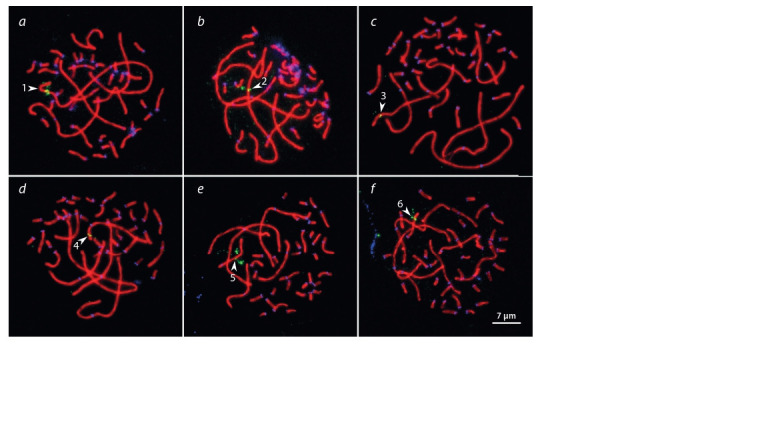
Pachytene spermatocytes of Pervomai roosters after immunolocalization SYCP3 (red), centromeric proteins (blue) and FISH with universal BAC probes (green) 184E5 (a), 44D16 (b), CH261-169K18 (c), CH261-83E1 (d ), CH261-2I23 (e), CH261-49F3 (f ). Arrowheads point to the SCs of the macrochromosomes identified by their sizes and centromeric indices.

In order to test the reliability of the morphological identification of macrochromosomes, we performed FISH with universal BAC probes obtained from the CHORY-261 library, marking chicken macrochromosomes, on SC preparations after immunolocalization of SYCP3 and centromeric proteins (Fig. 2). Comparison of the FISH results with the results of identification by relative sizes and centromeric indices showed good agreement for all chromosomes. We correctly identified the first seven macrochromosomes and chromosome Z. Chromosomes 6 and 7 are of similar SC lengths and are acrocentric and subacrocentric, respectively

**Fig. 3.**
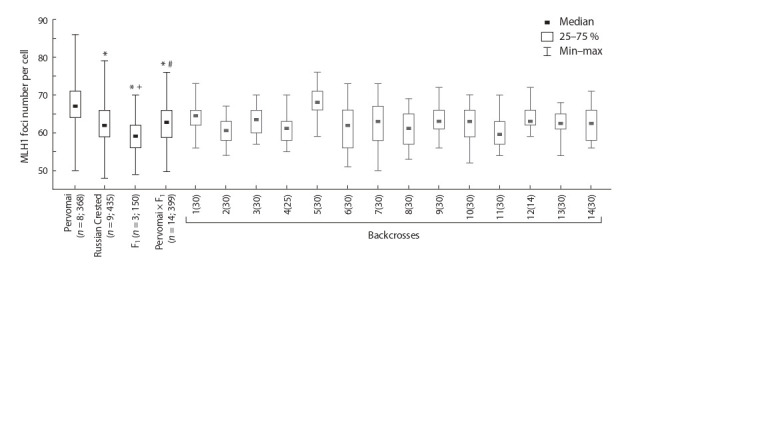
The number of MLH1 foci per spermatocyte in the roosters of two parental breeds, their F1 hybrids and backcrosses. The numbers in parentheses indicate the number of studied individuals and cells. Average values of genotypes are shown in black, individual values of backcrosses are shown in gray. "*" – differences with Pervomai, Mann–Whitney test, p < 0.01; "+" – differences with Russian Crested, Mann–Whitney test, p < 0.01; "#" – differences with F1, Mann–Whitney test, p < 0.01.

The average number of MLH1 foci per spermatocyte in the first generation hybrids (58.9 ± 0.3) was lower than in both parental breeds: Pervomai (67.3 ± 0.3) and Russian Crested (62.6 ± 0.3). The differences between hybrids and both parental breeds are significant (Mann–Whitney U-test is 11.4 and 14.2, respectively; p < 10–6). The backcrosses were homogeneous for the number of MLH1 foci (Fig. 3). They demonstrated a low average MLH1 foci number (62.6 ± 0.5), typical for the Russian Crested ( p = 0.80), although they exceeded F1 hybrids in this trait ( p <10–6). These results indicate negative heterosis of the recombination rate measured as MLH1 foci number per pachytene cell.

**Fig. 4.**
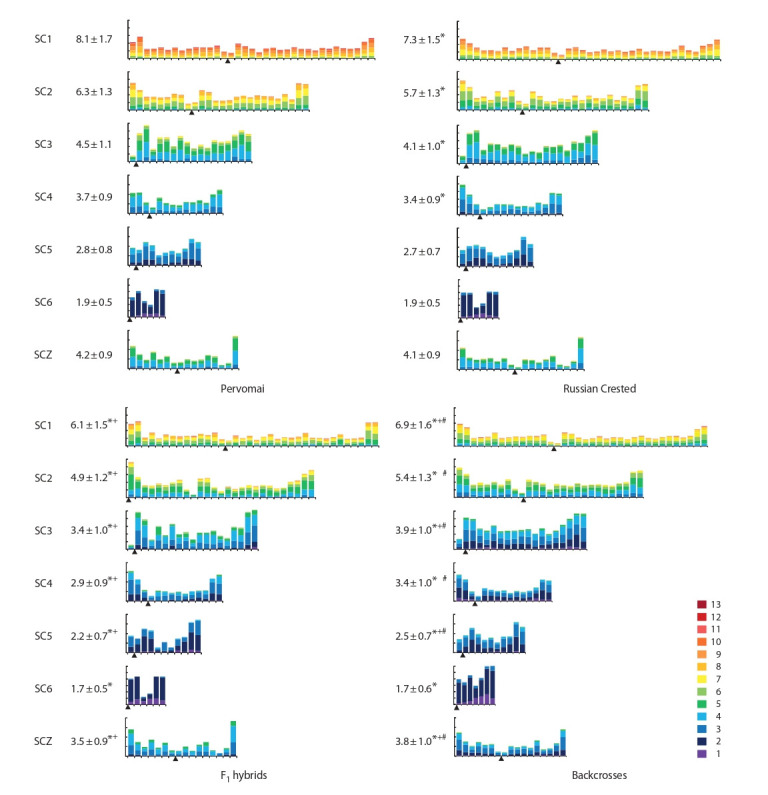
Average number and distribution of MLH1 foci along the macroSCs of Pervomai, Russian Crested roosters and their F1 hybrids and backcrosses. The X-axis ref lects the position of the foci in the bivalent relative to the centromere (indicated by a triangle). Each interval is equivalent to approximately 1 μm of the SC length. The Y-axis ref lects the proportion of nodules in each interval. Marks at the Y-axis in SC1–SC3 are equal to 0.02, in SC4–SC6, SCZ are equal to 0.05. The colors represent the proportion of bivalents with 1 to 13 MLH1 foci per chromosome. "*" – differences with Pervomai, Mann–Whitney test, p < 0.01; "+" – differences with Russian Crested, Mann–Whitney test, p < 0.01; "#" – differences with F1, Mann–Whitney test, p < 0.01.

These differences between parental breeds were mainly determined by the four largest macrochromosomes (Mann– Whitney test, p <0.01) (Fig. 4). SCZZ, SC5 and SC6 of the F1 hybrids contained fewer MLH1 foci than the corresponding macroSCs of the parental breeds, Pervomai and Russian Crested (Mann–Whitney test, p < 0.01). In the backcrosses to Pervomai, the number of MLH1 foci on the SC of ZZ and the six largest autosomes remained significantly smaller than on the corresponding SCs of Pervomai (Mann–Whitney test, p < 0.01). However, it was significantly higher on all SCs but SC6 than in the F1 hybrids (Mann–Whitney test, p < 0.01) (see Fig. 4).

Despite these differences in the number of MLH1 foci per particular macrochromosome between the parental breeds, F1 and backcrosses, each of them showed almost the same chromosome-specific pattern of MLH1 foci distribution along the SC (see Fig. 4). On most chromosomes, an increase in the frequency of recombination was observed in the distal regions

## Discussion

The most important and surprising result of our study is a discovery of overdominance of low recombination rate in F1 hybrids, measured as the number of MLH1 foci per pachytene cell. Backcrosses of the F1 hybrids to the parental breed with high recombination rate were rather homogenous and showed an intermediate recombination rate. Thus, the model of inheritance of recombination rate in roosters can be formally described as negative heterosis in F1 and additive inheritance in backcrosses.

The differences in overall recombination rate between the breeds, hybrids and backcrosses were mainly determined by the differences in the crossing over number in the large macrochromosomes. They are characterized by a high (up to 13!) and variable number of crossing overs, while small macrochromosomes have one or two chiasmata and each microchromosome contains only a single obligate chiasma necessary for orderly chromosome segregation

Generally, crossbreeds are expected to show positive heterosis for productivity traits (hybrid vigor) (Chen, 2013). This expectation contradicts the negative heterosis for the recombination rate observed in this study. Interestingly, the rate of dilution of heterosis for recombination rate in backcrosses is higher than the rate of dilution of positive heterosis for economic traits, at least in plants (Fridman, 2015). The decrease in recombination rate in F1 is probably determined by difficulties in homology matching between the DNA sequences of genetically divergent breeds (which we shall discuss below), rather than by dominant/overdominant genetic effects. With further level of backcrossing, the recombination rate acts like a regular complex trait with additive heritable component and environmental influence.

Our finding poses at least three interesting questions. How common is the negative heterosis for the recombination rate? What might be its molecular mechanism? What are its population genetic implications?

The first question is difficult to answer because we are aware of only a few prior studies in which recombination rates have been compared between parental breeds or species and their hybrids. There were no significant differences in autosomal recombination rate between two species of dwarf hamsters diverged about 1 MYA and their F1 female and male hybrids (Bikchurina et al., 2018). On the other hand, recombination in female hybrids between Microtus arvalis and M. levis diverged from 0.2 to 0.4 MYA and differing by a series of chromosomal rearrangements was significantly reduced compared to the parental species (Torgasheva, Borodin, 2016). Interspecific hybrids between Saccharomyces cerevisiae and S. paradoxus demonstrated low frequencies of genetic recombination (Hunter et al., 1996). Genome-wide introgression between two closely related nematode species Caenorhabditis briggsae and C. nigoni also revealed substantial suppression of recombination in the hybrids (Bi et al., 2015).

The molecular mechanism of negative heterosis for recombination rate is probably linked with the initial stages of chromosome synapsis and recombination, which includes scheduled generation of multiple double-strand DNA breaks (DSB), RAD51-mediated strand invasion and sequence homology matching (Zickler, Kleckner, 2015). Reduced recombination in interspecies hybrids may occur due to a significant decrease in homology between parent species accompanied by serious impairments of the chromosome synapsis in meiosis. However, even a minor decrease in homology at the early stages of divergence can apparently affect recombination due to decreased sequence identity. Comparison of recombination boundary sequences suggests that recombination in hybrids may require a region of high sequence identity of several kilobases in length (Ren et al., 2018).

Similarly, the study of recombination rate in hybrids between S. cerevisiae strains using high-throughput method showed a positive correlation of its level with sequence similarity between homologs at different scales (Raffoux et al., 2018). This is consistent with the finding that sequence divergence greater than about 1 % leads to the suppression of recombination due to heteroduplex rejection by the mismatch repair machinery (Chen, Jinks-Robertson, 1999). An antirecombination activity of the mismatch repair system during meiosis might contribute towards a decrease in recombination rate in hybrids between diverging breeds, populations and species (Radman, Wagner, 1993). At relatively low genetic distances it decreases the recombination rate in the hybrids, at greater genetic distances it impairs chromosome synapsis and might lead to hybrid sterility due to meiotic silencing of unpaired chromatin (Turner, 2015).

## Conclusion

There might be interesting evolutionary and population genetic implications of our findings. The negative heterosis for recombination in the hybrids may play an important role in speciation. Suppression of recombination impedes gene flow between parapatric populations and therefore accelerates their genetic divergence (Rieseberg et al., 1999; Baack, Rieseberg, 2007). A possibility of negative heterosis for recombination may also be taken into account in the calculations of the introgression time based on the size of linkage disequilibrium blocks (Payseur, 2010). They are based on the assumption that global and local recombination rates are constant over the generations. Our data indicate that it might not be the case. We detected a decrease in recombination in the macrochromosomes of the hybrids, while the microchromosomes retained the same recombination rate because it had already been the minimal required for orderly segregation.

## Conflict of interest

The authors declare no conflict of interest.
